# Pediatric diabetes update

**DOI:** 10.1111/1753-0407.13317

**Published:** 2022-09-16

**Authors:** Jasmine Gujral, Robert Rapaport

**Affiliations:** ^1^ Division of Pediatric Endocrinology Yale School of Medicine New Haven Connecticut USA; ^2^ Division of Pediatric Endocrinology and Diabetes Icahn School of Medicine at Mount Sinai New York New York USA; ^3^ Division of Pediatric Endocrinology and Diabetes Kravis Children's Hospital at Mount Sinai New York New York USA

## 1 STUDY DOES NOT SHOW AN ASSOCIATION BETWEEN DIABETES AUTOIMMUNITY AND SARS‐COV‐2 INFECTION IN CHILDREN

Multiple studies worldwide have shown an increased incidence of diabetes in children after COVID‐19 infection. One of the postulated mechanisms is triggering of an autoimmune response in the pancreatic beta cells. This hypothesis was tested in a study published in August 2022 in the *Journal of the American Medical Association* titled “SARS‐CoV‐2 Infections and Presymptomatic Type 1 Diabetes Autoimmunity in Children and Adolescents from Colorado, USA, and Bavaria, Germany.”^1^ In this cross‐sectional study, more than 50 000 children in Colorado, United States (aged 1–18 years, Autoimmunity Screening for Kids [ASK] study) and Bavaria, Germany (aged 1–10.9 years, Fr1da study) were screened in 2020 and 2021 for antibodies to SARS‐CoV‐2 infection and type 1 diabetes mellitus (T1DM). Antibodies to SARS‐CoV‐2 included antibodies to both receptor binding domain and nucleocapsid proteins and T1DM antibodies included autoantibodies to insulin, glutamic acid decarboxylase, islet antigen 2, and zinc transporter 8. Multivariable logistic regression was used to assess associations between previous SARS‐ CoV‐2 infection and islet autoimmunity

In Colorado, out of 4717 children, 32.3% (1524) had prior SARS‐CoV‐2 infection (median age, 8.6 years; 50.3% female). In Bavaria, out of 47 253 children, 6.1% (2862) were identified to have had a prior COVID‐19 infection (median age, 3.9 years; 48.9% female). Among 1524 children in Colorado, multiple islet autoantibodies for T1DM were detected in 21(0.45%) children and 26 (0.55%) children had a single islet autoantibody. Out of 2862 Bavarian children, 141 (0.30%) children had multiple T1DM antibodies whereas 54 (0.11%) children were positive for a single high‐affinity islet autoantibody. The prevalence of multiple or single high‐affinity islet autoantibodies did not significantly differ between children with or without previous SARS‐CoV‐2 infection in Colorado (1.18% vs. 0.91%, *p* = .43) or Bavaria (0.42% vs. 0.41%, *p* = .88). Therefore, previous SARS‐CoV‐2 infection was not significantly associated with the presence of multiple islet autoantibodies (odds ratio, 1.06 [95% confidence interval (CI), 0.59–1.80]; *p* = .83) or a single high‐affinity islet autoantibody (odds ratio, 1.34 [95% CI, 0.70–2.44]; *p* = .36). In Bavaria, 465 children were followed after the first detection of SARS‐CoV‐2 antibodies for a median of 8.9 months (up to 2 years); none developed islet autoantibodies

This study shows no significant association of previous COVID‐19 infection with the development of T1DM autoantibodies. However, longer follow‐up is needed to confirm these findings

## 2 NEW ONCE‐WEEKLY GLUCAGON‐LIKE PEPTIDE‐1 (GLP‐1) RECEPTOR AGONIST SHOWS PROMISING RESULTS FOR YOUTH WITH TYPE 2 DIABETES

GLP‐1 receptor agonists liraglutide (once daily) and exenatide (once weekly) have recently been approved for the treatment of youth with type 2 diabetes. In a study published in the *New England Journal of Medicine* in August 2022, dulaglutide, a once weekly GLP‐1 receptor agonist, was studied for efficacy and safety in a phase 3, randomized, double‐blind, placebo‐controlled trial, Assessment of Weekly Administration of LY2189265 in Diabetes–Pediatric Study^2^ in Youth with Type 2 Diabetes ^3.^ This multicenter trial was conducted in 46 centers in nine countries and participants were enrolled between December 2016 and December 2020. Inclusion criteria were children between ages 10–18 years, a body‐mass index (BMI) greater than the 85th percentile for age and sex, and a glycated hemoglobin level between 6.5% to 11.0%. The participants could be on metformin and insulin, but their doses had to be stable (defined as varying within 15%).

A total of 154 participants were enrolled and underwent randomization into three groups: once‐weekly treatment with dulaglutide at a dose of 0.75 mg, once‐weekly treatment with dulaglutide at a dose of 1.5 mg, or placebo. The double‐blinded period was 26 weeks, followed by a 26‐week open‐label extension period, where participants who had been assigned to a dulaglutide group continued to receive open‐label dulaglutide at their assigned dose, and participants who had been assigned to the placebo group began receiving open‐label dulaglutide at a dose of 0.75 mg weekly.

The primary end point of this study was the change in the glycated hemoglobin level from baseline to week 26, which showed a significant reduction by 0.8 percentage points in the pooled dulaglutide groups but increased by 0.6 percentage points in the placebo group (estimated treatment difference of −1.4 percentage points; 95% CI, −1.9 to −0.8; *p* < .001). The glycated hemoglobin level tended to increase during the open‐label period, speculated to be from rapid underlying disease progression. 65% of those receiving the 0.75‐mg dose and 55% of those^3^ Reductions in the fasting blood glucose concentration in the dulaglutide groups were sustained through 52 weeks. However, dulaglutide was not superior to placebo in lowering BMI. Adverse effects were mild—most commonly nausea (80%), vomiting (69%), and diarrhea (89%)—and mostly transient, occurring during the first 2 weeks of initiation of dulaglutide therapy.

Dulaglutide, when approved for pediatric diabetes, would be beneficial not only for glycemic control but would also be popular given its once‐weekly frequency.

## 3 TESTING FOR TYPE 1 ANTIBODIES AT AGE 2 AND 6 YEARS PROVIDES GOOD SENSITIVITY AND SPECIFICITY FOR PREDICTING ONSET OF DIABETES BY AGE 15 YEARS

Islet autoantibodies and HLA genotyping can help in predicting future risk of T1DM in children; however, the time between detection of antibodies and clinical presentation of T1DM is highly variable. In this large prospective study, data were combined from four countries and five cohorts from Finland, Germany, Sweden, and the United States to study 24 662 children at high risk of T1DM. The children were deemed high risk either based on strong family history of first‐degree relative with T1DM or HLA genotyping. Plasma was screened using islet autoantibodies against glutamic acid decarboxylase, insulinoma antigen 2, and insulin. Zinc transporter 8 antibodies were not universally measured and therefore not included in the analysis. The first screening was done by age 2.5 years and annual screening for autoantibodies and diabetes was done from age 1 to up to age 15 or T1DM onset, whichever occurred first. Main outcomes tested were sensitivity and positive predictive value (PPV) of detected islet autoantibodies, tested at one or two fixed ages, for diagnosis of clinical T1DM.

Of the 24 662 participants enrolled, 6722 were followed up to age 15 years or until onset of T1DM. Out of the 6722 children, 672 developed T1DM by age 15 years. Results showed that screening at two ages was better than screening at one age for prediction of T1DM. The optimal age for a single screening was 4 years, but for a two‐age screening the best ages to screen were 2 years and 6 years offering a sensitivity of 82% (95% CI, 79–86) and PPV of 79% (95% CI, 75–80) for onset of diabetes by age 15 years. Autoantibodies usually appeared before age 6 years in children diagnosed with diabetes much later in childhood. These results show that an initial screening for islet autoantibodies at two ages (2 and 6 years) is an efficient strategy for T1DM prediction by age 15 years.

However, the overall prevalence of autoimmunity for T1DM is quite low. This is evidenced by a population study in Bavaria, Germany where 154 462 children aged 2–10 years were enrolled in the Fr1da public health screening program for T1DM.^4^ Out of this cohort, only 541 participants (0.35%) screened positive of whom only 0.08% (119) were positive for T1DM. Therefore, the population prevalence numbers show that the path forward requires we develop effective treatment approaches post diagnosis.
